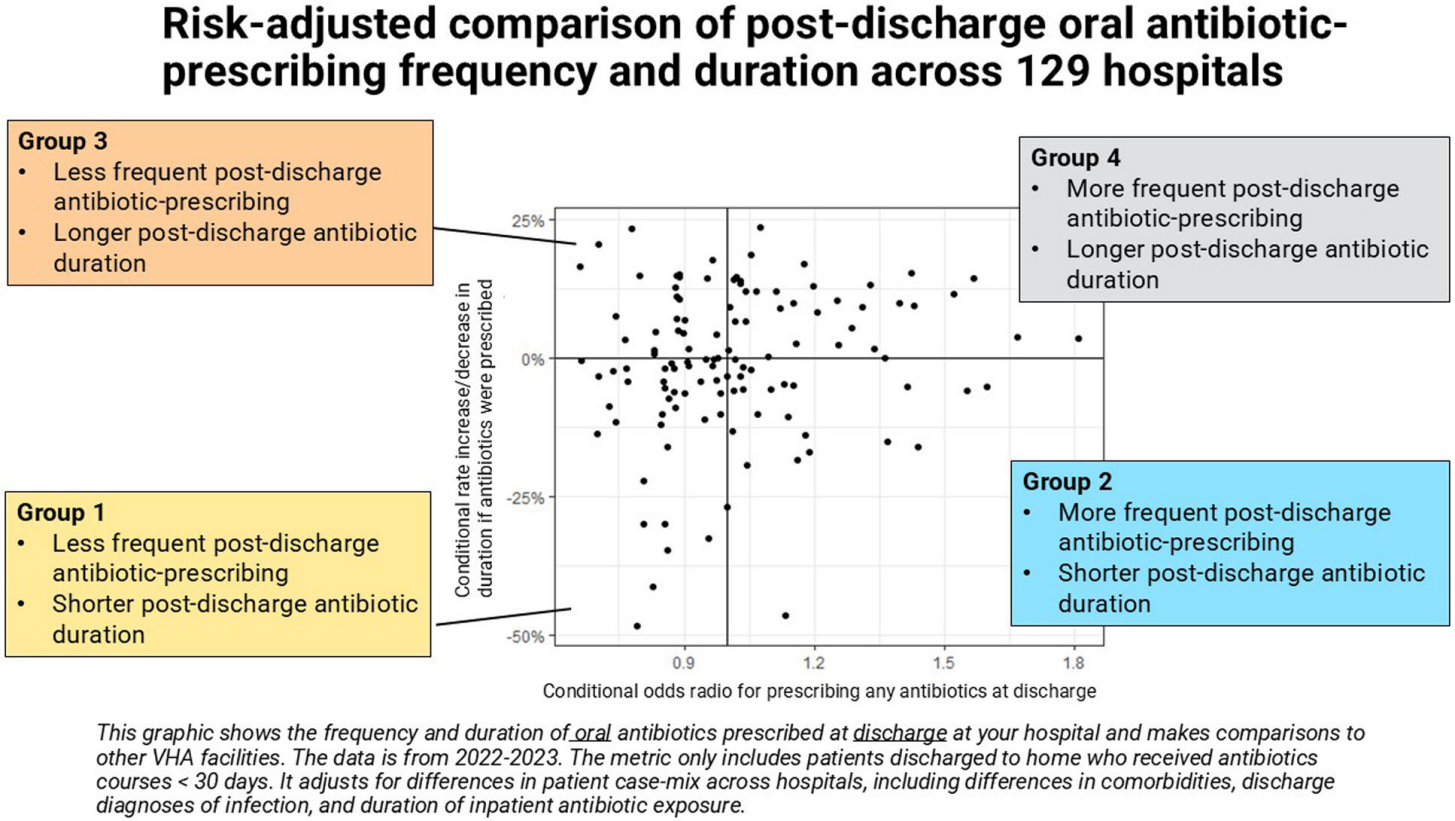# 70 Clinical Impact of Candida Growth in Organ Preservation Fluid: A Retrospective Analysis of Abdominal Solid Organ Transplants

**DOI:** 10.1017/ash.2026.10500

**Published:** 2026-06-23

**Authors:** Brittany Anderson, Stacey Hockett Sherlock, Nicole Johnson, Kimberly Dukes, Daniel Livorsi

**Affiliations:** 1 Center for Access and Delivery Research and Education (CADRE) VA Iowa City Health Care System; 2 University of Iowa, Carver College of Medicine; 3 Iowa City VA Health Care System; 4 Dept of Gen Int Med, Carver College of Medicine, University of Iowa; 5 University of Iowa, Department of Internal Medicine

## Abstract

**Background:** Antibiotic overuse is common at hospital discharge. Antibiotic stewardship [ASP] metrics that capture antibiotic-prescribing at discharge can identify opportunities to optimize prescribing. We conducted a qualitative analysis of clinician perceptions of a novel risk-adjusted metric that shows the frequency and duration of oral antibiotics prescribed at discharge at Veterans Health Administration (VHA) hospitals. **Methods:** We conducted 91 semi-structured interviews with clinicians (e.g., ASP champions, hospitalists, pharmacists) from nine VHA facilities. Facilities represented three performance groups on the metric [Figure 1]: Group 1: less frequent antibiotic prescribing at discharge and shorter duration; Group 3: less frequent and longer duration; Group 4: more frequent and longer duration. During the interview, we presented the metric and explained its design and the facility's placement on the metric. Our interview guide prompted responses concerning clarity, fairness, utility of the metric for stewardship goals, and thoughts on the metric broadly. We conducted thematic analysis on interview transcripts. **Results:** The majority of clinicians from each performance group reported that the metric made sense and conveyed results clearly, expressing that the metric could support stewardship practices by reinforcing appropriate prescribing and identifying areas for improvement. Yet, many clinicians highlighted that they needed additional granular data to inform effective interventions, including service-specific prescribing, which infections are being overtreated, and how close their hospital is to being categorized in a different performance group. Clinicians often proposed a potential explanation for why their facility was in a specific performance group, including descriptions of strong ASP programs or reflections on patient complexity or services that may drive more frequent or longer prescribing. Responses on fairness varied across performance group and clinician role. Clinicians voiced concerns about the possible punitive use of the metric without considering whether it aligned with the appropriateness of antibiotic prescribing. Clinicians wanted more information about how the metric accounted for differences in patient case-mix. **Conclusions:** Our study found that clinicians generally considered the novel metric for comparing antibiotic prescribing at VHA hospital discharges to be clear and useful. This metric has potential to strengthen antibiotic stewardship by identifying areas needing improvement. However, clinicians emphasized the need for more granular data and raised concerns about fairness and potential punitive use. Addressing these points and clearly framing the metric as a tool for quality improvement will be essential for successful implementation.

**Figure:**